# Prenatal muscle development in a mouse model for the secondary dystroglycanopathies

**DOI:** 10.1186/s13395-016-0073-y

**Published:** 2016-02-19

**Authors:** Jihee Kim, Mark Hopkinson, Manoli Kavishwar, Marta Fernandez-Fuente, Susan Carol Brown

**Affiliations:** Department of Comparative Biomedical Sciences, Royal Veterinary College, University of London, London, UK

**Keywords:** Dystroglycanopathy, Pax7, Congenital muscular dystrophy, Fukutin-related protein, Laminin, Dystroglycan

## Abstract

**Background:**

The defective glycosylation of α-dystroglycan is associated with a group of muscular dystrophies that are collectively referred to as the secondary dystroglycanopathies. Mutations in the gene encoding fukutin-related protein (FKRP) are one of the most common causes of secondary dystroglycanopathy in the UK and are associated with a wide spectrum of disease. Whilst central nervous system involvement has a prenatal onset, no studies have addressed prenatal muscle development in any of the mouse models for this group of diseases. In view of the pivotal role of α-dystroglycan in early basement membrane formation, we sought to determine if the muscle formation was altered in a mouse model of FKRP-related dystrophy.

**Results:**

Mice with a knock-down in FKRP (FKRP^KD^) showed a marked reduction in α-dystroglycan glycosylation and reduction in laminin binding by embryonic day 15.5 (E15.5), relative to wild type controls. In addition, the total number of Pax7^+^ progenitor cells in the FKRP^KD^ tibialis anterior at E15.5 was significantly reduced, and myotube cluster/myofibre size showed a significant reduction in size. Moreover, myoblasts isolated from the limb muscle of these mice at E15.5 showed a marked reduction in their ability to form myotubes in vitro.

**Conclusions:**

These data identify an early reduction of laminin α2, reduction of myogenicity and depletion of Pax7^+^ progenitor cells which would be expected to compromise subsequent postnatal muscle growth and its ability to regenerate postnatally. These findings are of significance to the development of future therapies in this group of devastating conditions.

## Background

Dystroglycan is a key component of the dystrophin-glycoprotein complex (DGC) which connects the extracellular matrix to the muscle fibre cytoskeleton [1]. It is composed of two non-covalently associated sub-units, α and β. α-dystroglycan is subject to extensive O-mannosylation which is responsible for binding to extracellular matrix proteins such as laminin, perlecan and agrin [2], in addition to neurexin in the brain [3], pikachurin in the eye [4] and most recently, slit [5] in the nervous system. β-dystroglycan is a transmembrane protein which links α-dystroglycan to dystrophin and utrophin on the cytoplasmic side of the sarcolemma [6]. The abnormal glycosylation of this protein is the underlying cause for a subset of inherited autosomal recessive muscular dystrophies, collectively known as the secondary dystroglycanopathies [7, 8]*.*

To date, up to 18 genes have been implicated in the secondary dystroglycanopathies. Mutations in the fukutin-related protein (*FKRP*) gene are a common cause of secondary dystroglycanopathy and are associated with a wide range of clinical features, including severe structural brain involvement resembling Walker–Warburg syndrome (WWS) and muscle-eye-brain disease (MEB), to congenital muscular dystrophy without brain involvement (MDC1C) and adult-onset limb-girdle muscular dystrophy (LGMD2I) [9–23]. LGMD2I represents the most common form of LGMD in the UK [8]. Phenotypic variability is not unique for mutations in the *FKRP* gene, and a similar spectrum of clinical involvement has been observed for most of the other dystroglycanopathy genes [24].

Muscle differentiation is accompanied by marked changes in the glycosylation of α-dystroglycan [25, 26] mediated at least in part by the coordinated upregulation of like-acetylglucosaminyltransferase (LARGE), a bifunctional glycosyltransferase which is known to be responsible for the post-translational addition of the polysaccharide repeating unit [-3-xylose-α1,3-glucuronic acid-β1-] on α-dystroglycan [27]. Blocking this process leads to a less compact basement membrane, immature neuromuscular junctions and abnormal muscle predisposed to dystrophy [27]. However, the impact of altered/reduced glycosylation of α-dystroglycan on prenatal muscle development has not yet been evaluated despite the implications that this might have for muscle mass in dystroglycanopathy patients [28].

Myogenesis in the mouse may be divided into two main stages; the first is referred to as primary (embryonic) myogenesis. During this phase, primary myotubes are generated through myoblast proliferation and fusion. The primary myotubes make an appearance between E11 and E14 in mice, extending from tendon to tendon, and the majority initially expresses the slow myosin heavy chain (MHC) although expression may switch later depending on the individual muscle [29]. Groups of primary myotubes are initially surrounded by a single basement membrane but subsequently separate and become surrounded by their own basement membrane [30]. The primaries act as a scaffold for subsequent generations of myoblasts that fuse to form secondary, and in the larger muscles, tertiary myotubes [30]. The secondary myogenesis in the mouse takes place between E14.5 and E17.5. The early secondary myotubes initially form close to the neuromuscular junctions of the primary [31]. The majority of fibres in the adult muscle originate from the secondary and tertiary myotubes, and final fibre number is determined around the time of birth [30].

We previously generated a *FKRP-*deficient mouse which has a knock-down in the expression of the *FKRP* gene (FKRP^KD^). These mice die around the time of birth or within the first 24 h due to central nervous system involvement [32, 33] since restoration of *FKRP* in all neural tube-derived cells prevents perinatal lethality [34]. In the present work, we used the FKRP^KD^ mouse to examine the role of α-dystroglycan glycosylation on primary and secondary myogenesis. On the basis of the previous work which suggests that a deficiency of fukutin is associated with an impairment of myoblast precursor cell proliferation, differentiation and muscle regeneration [35], our hypothesis was that a reduction in α-dystroglycan glycosylation would be associated with a reduction in Pax7^+^ progenitor cells. Since FKRP^KD^ mice are not associated with an overt muscle pathology at birth [32], we further hypothesized that the initial divergence between FKRP^KD^ and wild type would take place during the later stages of muscle development. An evaluation of the tibialis anterior (TA) and extensor digitorum longus (EDL) at E15.5 and P0 (at birth) indicated that both these muscles showed a reduction in α-dystroglycan glycosylation and laminin binding in the FKRP^KD^ compared to wild types. Moreover, there was a significant reduction in myotube cluster size at E15.5 in the TA, implying that the secondary myogenesis was altered. This was further supported by the finding that there was a reduction in the myogenicity of cultures derived from the mice at this age. Associated with this was a significant reduction in the number of Pax7^+^ progenitor cells at E15.5 and P0 in the TA. Whilst differences were also noted in the EDL, the data did not achieve statistical significance. Indeed, this might be expected given that individual muscles often differ with respect to their stage of development during embryogenesis. Overall, these findings suggest that defects in α-dystroglycan glycosylation are associated with an early reduction in the number of Pax7^+^ progenitor cells which would be expected to compromise subsequent postnatal muscle growth and regeneration.

## Methods

### Mice

All animal experiments were carried out under license from the Home Office (UK) in accordance with The Animals (Scientific Procedures) Act 1986 and were approved by the Royal Veterinary College ethics and welfare committee. The FKRP-^NeoTyr307Asn^ (FKRP^KD^) has a neomycin selection cassette in intron 2 and a missense mutation in exon 3. The missense mutation (Tyr307Asn) has previously been shown to result in no discernable phenotype in the mouse; however, the presence of the neomycin selection cassette results in a 60–80 % reduction in *FKRP* transcript levels [32, 36]. The morning of identification of the copulatory plug was taken to be E0.5. On E15.5 of gestation, the dam was euthanised by atlanto-occipital dislocation. The embryos were then dissected out of the uterus and together with pups at P0 sacrificed by cervical dislocation. Tail samples from each were collected and retained for genotyping. The hindlimbs from at least five embryos/pups in each group were dissected, mounted in OCT and snap-frozen in liquid nitrogen-cooled isopentane. Generally, all measurements were undertaken on the same embryos/pups; however, limited quantities of the tissue sometimes necessitated additional embryos/pups being included in the analyses; hence, the *n* values differ between some data sets. All tissue was stored at −80 °C prior to sectioning with a Bright cryostat. Serial 10 μm cryosections were taken from the entire lower hindlimb and sections of the midbelly region of the EDL and TA selected for immunolabelling.

### Genotyping

Genotyping was carried out by PCR using three primers. Primers: FKRP forward (GTTGTGCTTAAACCACCTTC) and FKRP reverse (CTAGGAGGTTGAGGATGATGG) were used for detecting the wild type allele, and FKRP-Neo forward (GGTGGGATTAGATAAATGCC) and FKRP reverse were used for detecting the mutant allele.

### Alcian blue/alizarin red staining

Alcian blue stains the cartilage (blue) whilst alizarin red stains the ossified bone (red). Newborn (P0) pups were culled by cervical dislocation, and the skin, viscera and adipose tissue were removed. Bodies were placed in acetone for 2 days and then stained with 0.3 % (*w*/*v*) alcian blue 8GS in 70 % ethanol (1 volume), 0.1 % (*w*/*v*) alizarin red S in 95 % ethanol (1 volume), acetic acid (1 volume) and 70 % ethanol (17 volumes). Filtered stock solutions of alizarin red and alcian blue were made in advance. The following staining samples were washed in distilled water and then cleared in 1 % (*w*/*v*) solution of potassium hydroxide for 12–48 h. Once the skeleton was clearly visible, samples were cleared with 20, 50 and 80 % glycerol in 1 % potassium hydroxide. Once cleared, all samples were stored in 100 % glycerol.

### Measurements of tibia and femur length by CT scan

Tibia and femur length was measured using micro-computed tomography data of scanned newborns. Newborn (P0) pups underwent 180° scans in a Skyscan 1172F scanner with a 10 MP X-ray detector (Bruker, Kontich, Belgium). The X-ray source settings were at 40 kV and 200 mA, a 0.5-mm aluminium filter was used with a 6-μm pixel size and an exposure time of 2300 ms. Projection images were reconstructed into tomograms using NRecon (Skyscan) and then volume rendered using CTVox (Skyscan) to give 3D models which were measured using ImageJ [37].

### Immunohistochemistry

Prior to immunolabelling, all slides were allowed to air-dry. Unfixed sections were used throughout this study with the exception of the Ki67 (DAKO), Pax7 (DSHB) and IIH6 (Millipore) where sections were fixed in 4 % paraformaldehyde (PFA) for 10 min, washed and permeabilised in 0.1~0.5 % triton in phosphate-buffered solution (PBS) for 10–15 min. After washing, sections were blocked in 10 % horse serum (PAA) in PBS (10 % horse serum in PBS + 5 %BSA for IIH6) for 1 h prior to incubation with adequate primary antibodies at room temperature for 1 h (with the exception of the IIH6 antibody which was incubated overnight at 4 °C). To minimise background staining when labelling for Pax7 and Ki67, the Mouse On Mouse immunodetection kit was used (Vector Laboratories). All the other primary antibodies (listed in Table 1) were applied to the sections after blocking in 10 % horse serum in PBS for 1 h at room temperature. All dilutions were made up with PBS. The primary antibodies were incubated for 1 h at room temperature and the secondary antibodies for 30 min at room temperature followed by another 15 min incubation with streptavidin 488/594 (room temperature, Invitrogen) if a biotinylated secondary antibody was used. Nuclei were labelled with Hoechst 33342 trichloride (DAPI) (Sigma-Aldrich), which was diluted at 1:2000 with PBS. Controls for background included an incubation with the secondary antibody. In between all incubations, slides were washed in PBS three times for 5 min each time. The slides were then mounted with cover slides using Hydromount (Diagnostic laboratories, UK). Immunolabelled slides were viewed under epifluorescent microscope (Leica DM4000B interfaced with a Zeiss AxioCam MRm monochrome camera.

**Table 1:** Antibodies used in immunohistochemistry

Antibodies	Manufacturer	Dilution	Species
α-dystroglycan (IIH6)	Millipore	1:200	Mouse (IgM)
β-dystroglycan	Vector laboratories	1:20	Mouse
Pax7	Developmental Studies Hybridoma Bank (DSHB)	1:50	Mouse
Ki67	DAKO	1:200	Rat
Type IV collagen	Invitrogen	1:50	Goat
Perlecan	Invitrogen	1:5000	Rat
Pan Laminin	Sigma	1:200	Rabbit
Laminin α1	Gift from Professor Madeleine Durbeej-Hjalt	1:100	Rat
Laminin α2	Enzo	1:200	Rat
Laminin α4	R&D systems	1:200	Goat
Laminin α5	Gift from Madeleine Durbeej-Hjalt	1:200	Rabbit
Laminin β1	Millipore	1:2000	Rat
Laminin γ1	Millipore	1:2000	Rat
Fast myosin heavy chain	Leica	1:20	Mouse
Slow MHC	Millipore	1:200	Mouse
Developmental MHC	Leica	1:20	Mouse
Mandys1 (dystrophin)	Gift from Professor Glenn Morris	1:20	Mouse
Myogenin	Santa Cruz	1:200	Rabbit
MyoD	Santa Cruz	1:200	Rabbit

### Western blotting and laminin overlay assay

The muscle was dissected from the entire limb and cell proteins extracted in lysis buffer, radio immunoprecipitation assay (RIPA) buffer consisting of 50 mM Tris-HCl pH 7.5, 150 mM NaCl, 1 mM EDTA, 1 % Triton X-100, 0.1 % SDS and 1 mM azide, plus a cocktail of protease inhibitors (Roche). Protein concentration was measured using the BCA Protein Assay Kit (Thermo Scientific Pierce). A 30 μg of soluble proteins was resolved using a NuPAGE Pre-cast gel (3–8 % Tris-acetate; Invitrogen) and then transferred electrophoretically to Hybond-PVDF membrane (GE Healthcare, UK). PVDF membrane were blocked in 5 % dried non-fat milk in phosphate-buffered saline buffer, and then probed with the primary antibodies: anti-mouse α-DG IIH6 (Millipore, UK, cat, 05-593) anti-mouse β-DG (Vector Labs, UK), at room temperature for 1 h. After washing, they were incubated with the appropriate horseradish peroxidase (HRP)-conjugated secondary antibody for 1 h: anti-mouse-IgM or anti-mouse-IgG (both from Jackson ImmunoResearch, USA). After washing, the membranes were visualized using enhanced chemiluminescence (ECL) substrate (Thermo scientific Pierce) and imaged using a Bio-Rad ChemiDoc MP imaging system.

For the laminin overlay assay, PVDF membranes were blocked for 1 h in laminin-binding buffer (LBB; 10 mM triethanolamine, 140 mM NaCl, 1 mM MgCl2, 1 mM CaCl2, pH 7.6) containing 5 % non-fat dry milk followed by incubation of 1 μg/ml mouse Engelbreth–Holm–Swarm (EHS) laminin protein (Invitrogen) in LBB overnight on a roller at 4 °C. The membranes were washed and incubated with rabbit anti-laminin (Sigma) followed by HRP-anti-rabbit IgG (Jackson ImmunoResearch). Blots were visualized using enhanced chemiluminescence (ECL) substrate (Thermo scientific Pierce) and imaged using a Bio-Rad ChemiDoc MP imaging system.

### Tissue culture

All muscles were removed from the limbs of E15.5 embryos and digested with freshly made enzyme solution. The enzyme solution consisted of phosphate-buffered saline (PBS; Gibco) mixed with 0.5 mg/ml *w*/*v* collagenase (Sigma–Aldrich), 1 mg/ml *w*/*v* Bovine serum albumin (BSA; Sigma–Aldrich) and 6 % *v*/*v* Trypsin-ethylene diamine tetra acetic acid (EDTA; Invitrogen). This was incubated with the muscle following trituration for 30 min at 37 °C. After 30 min, the enzyme solution was removed and discarded in order to dispose of fibroblasts. The second and third digestions were then undertaken, and the cells from each were pooled and placed in growth medium (GlutaMAX DMEM (Gibco)) containing 1 % penicillin–streptomycin–neomycin (PSN; Gibco) and 20 % fetal bovine serum (FBS). This cell solution was then plated onto Matrigel (1 mg/ml) coated plate or chamber slides directly to facilitate proliferation. Differentiation was initiated by switching to 10 % FBS after 48 h (Gibco).

### Quantification and statistical analysis

ImageJ was used to measure myotube clusters/fibre size [37]. This was done manually using a light pen on images (resolution 150 pixels/in.) at either ×10 or ×20 magnification.

The expression of the transcription factor Pax7 was assessed across the entire cross-sectional area of the muscle. The quantification of the number of Pax7^+^ nuclei was expressed as the number per fibre in the TA and EDL at E15.5 and P0.

Data were analysed using GraphPad Prism 5 software. Confirmation of normality was undertaken using Kolmogorov–Smirnov test; unpaired *t* tests were performed to compare wild type and mutant animals and ANOVA for comparisons between wild type, heterozygotes and mutants.

## Results

### Litter size and longitudinal growth were not altered in the newborn FKRP^KD^ mice

**Fig. 1:**
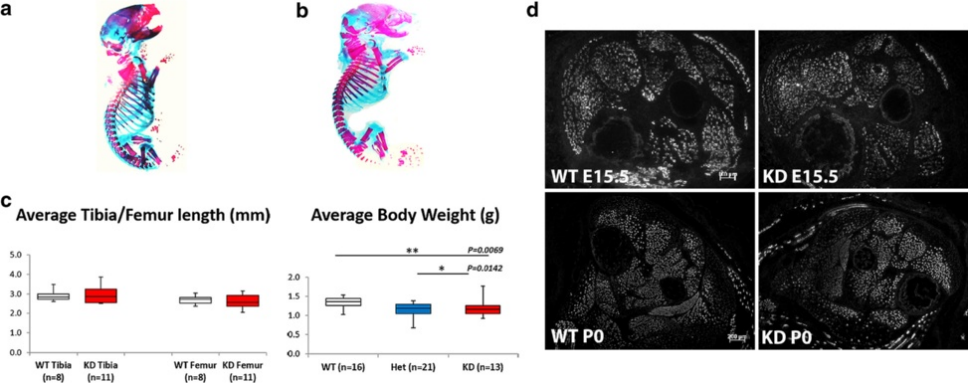
Skeletal development, body weight and skeletal muscle development. A comparison of alizarin blue staining of wild type (**a**) and FKRP^KD^ (**b**) pups at P0 indicated no differences with respect to skeletal development. **c** Body weight was significantly lower in FKRP^KD^ (*p* = 0.0069) relative to wild type; there was no significant difference in body weight between wild type and heterozygous mice. Tibia/femur length was measured using micro-computed tomography and showed no significant differences between FKRP^KD^ and wild type at P0. Data was analysed by one-way ANOVA. **d** Transverse sections of the hindlimb muscle at E15.5 and P0 immunolabelled for slow myosin heavy chain. The proportion of myotubes expressing slow myosin heavy chain was similar between FKRP^KD^ and wild type. At P0, fewer myotubes/fibres expressed slow myosin, with those still positive distributed in the superficial area of the TA and EDL. In the soleus which is a slow postural muscle in the adult, slow myosin continued to be expressed in most of myotubes/fibres

A comparison of alcian blue/alizarin red-stained wild type and FKRP^KD^ pups at P0 which showed the extent of the ossified bone (red) and cartilage (blue) indicated no significant differences with respect to skeletal development between the two genotypes (Fig. 1a, wild type and 1B FKRP^KD^). Measurements of the tibia and femur using computed tomography as an indirect measurement of muscle length showed no significant differences between WT and FKRP^KD^ (Fig. 1c). Body weight was significantly lower in the FKRP^KD^ at P0 relative to wild type as previously reported, whilst litter size influenced this parameter, overall litter size was not significantly different between WT and FKRP^KD^.

### A reduction in α-dystroglycan glycosylation does not alter muscle patterning but is associated with an alteration in laminin immunobinding by E15.5 and P0

**Fig. 2:**
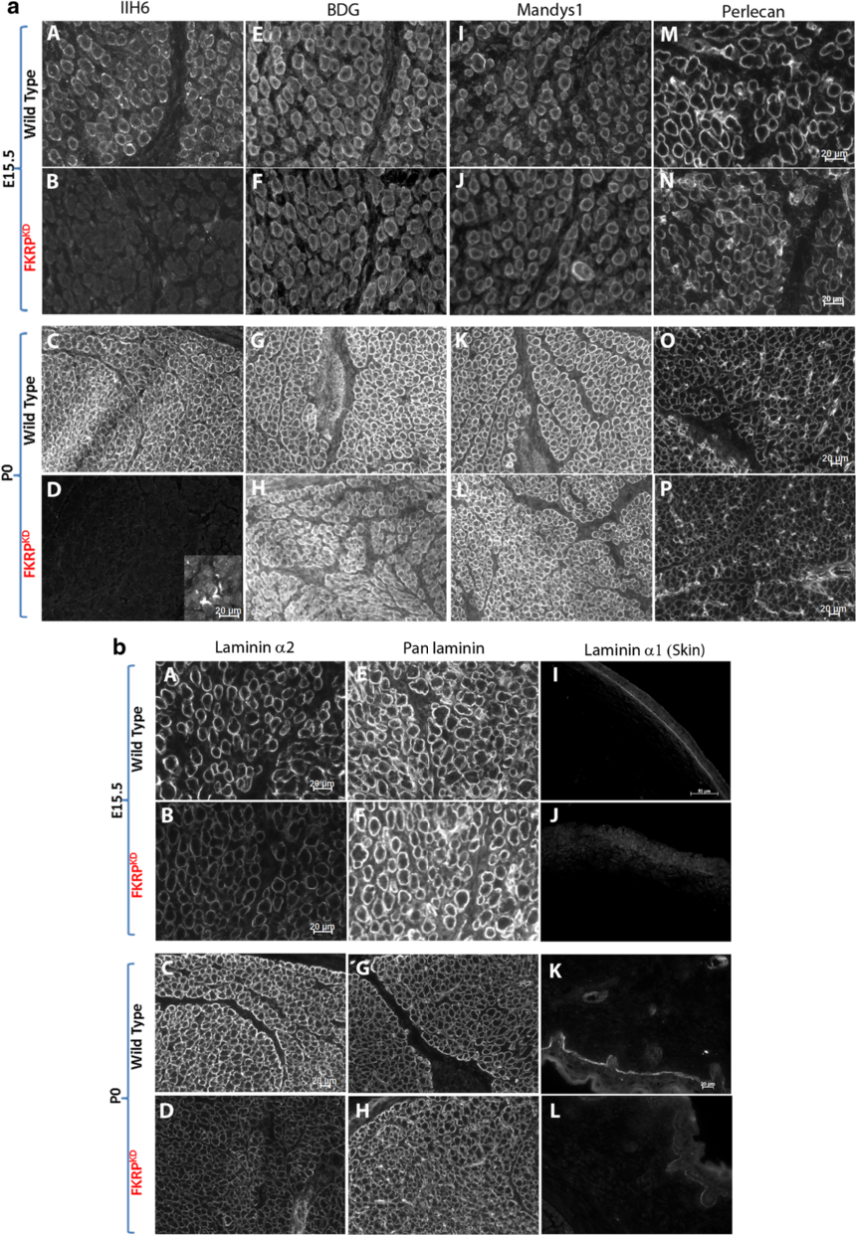
Immunolabelling of α- and β-dystroglycan, dystrophin and perlecan. **a** Transverse sections through the TA and EDL at E15.5 and P0 immunolabelled for α- (A-D) and β-dystroglycan (E-H), dystrophin (Mandys1, I-L) and perlecan (M-P). α-dystroglycan (IIH6) showed a clear delineation of the basement membrane around clusters of primary and secondary myotubes at E15.5 (A) and individual muscle fibres at P0 (C), although some clusters are still evident. There was an almost total absence of IIH6 in the FKRP^KD^ at both time points (B,D). Residual staining of the neuromuscular junction in the FKRP^KD^ at P0 is shown in D as an inserted image. β-dystroglycan, dystrophin and perlecan immunolabelling were not markedly different between FKRP^KD^ and wild type at either time point (E-P)

Transverse sections through the entire lower hindlimb at E15.5 and P0 of both FKRP^KD^ and wild type immunolabelled for slow myosin heavy chain showed no alteration in muscle patterning (Fig. 1d). In the tibialis anterior (TA) and extensor digitorum longus (EDL), a population of the larger primary myotubes could be clearly identified. The majority of these primaries at E15.5 were surrounded by smaller secondaries forming myotube clusters which were enclosed within a single basement membrane delineated by immunolabelling for laminin, perlecan or collagen type IV. In wild type mice immunolabelling for IIH6 (identifies the laminin binding epitope of α-dystroglycan) followed that of other basement membrane markers (Fig. 2a). However, in the FKRP^KD^ at E15.5 and P0, although there was some variation between pups most likely due to the model being a hypomorph, overall, there was a marked reduction in IIH6 immunolabelling. These observations therefore indicate that glycosylated α-dystroglycan is present from the earliest stages of myotube formation in wild type mice and is markedly reduced in the FKRP^KD^. However, β-dystroglycan was unchanged between wild type and FKRP^KD^ as was dystrophin which marks the sarcolemma of both primary and the early secondaries (Fig. 2a). Perlecan, an additional ligand of α-dystroglycan also dependent on its proper glycosylation showed no difference between mutant and wild type (Fig. 2a).

**Fig. 3:**
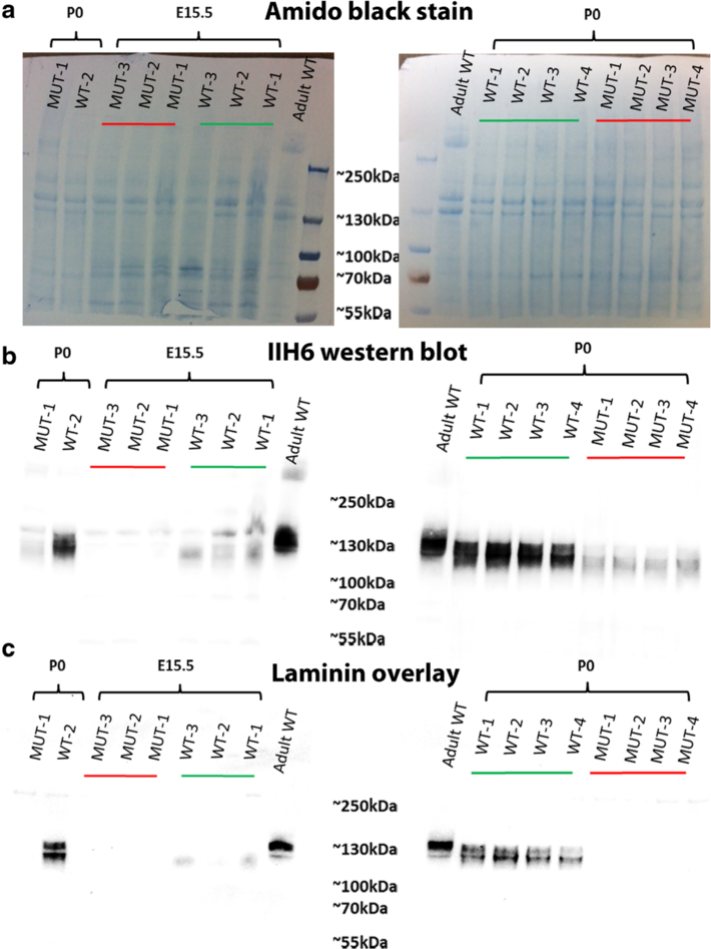
IIH6 Western blot and laminin overlay at E15.5 and P0. **a** Shows an amido black stain of the transfer membrane indicating equivalent loading in all lanes. **b** Western blot for IIH6 in the hindlimb muscle at E15.5 and P0, the relative molecular mass of α-dystroglycan in the wild type is reduced compared to the adult mouse muscle and only residual IIH6 labelling was observed in FKRP^KD^. **c** At both E15.5 and P0, α-dystroglycan was able to bind laminin in the wild type and no binding was observed in the FKRP^KD^

In order to make an assessment of the relative molecular mass and laminin binding properties during muscle develoImmunolabelling using an antibodypment, we next compared α-dystroglycan at E15.5 and P0 in wild type and FKRP^KD^ mice using Western blotting. This showed that, as has been previously observed during development of the chick and human muscle, the relative molecular mass of α-dystroglycan in wild type muscle is reduced in fetal relative to the adult mouse muscle (Fig. 3b) [25, 26]. Protein loading was assessed using Amido Black staining of the PVDF membrane (Fig. 3a). In agreement with the immunolabelling, only residual IIH6 labelling was observed following Western blot analysis of FKRP^KD^ at either E15.5 or P0. A laminin overlay assay further showed that whilst α-dystroglycan was able to bind laminin at both E15.5 or P0 in the wild type, no binding was observed in the FKRP^KD^ (Fig. 3c). Taken overall, these observations imply that α-dystroglycan glycosylation plays a role in basement membrane assembly during the early stages of fibre formation.

### Expression of laminin α2 is reduced early during secondary myogenesis

**Fig. 4:**
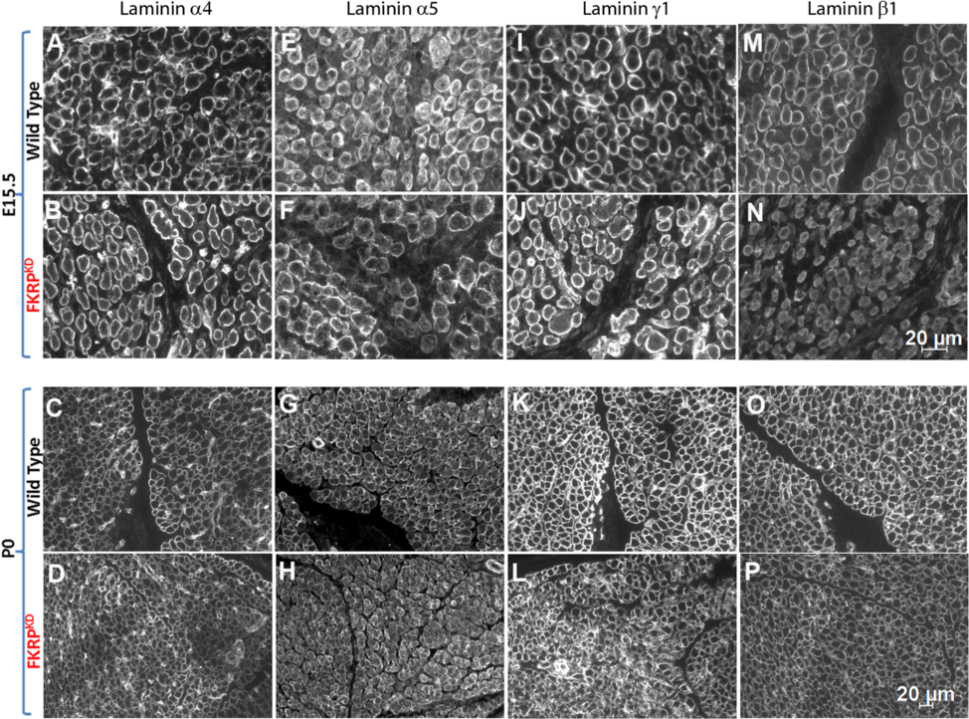
Immunolabelling of laminins α4, α5 γ1 and β1 at E15.5 and P0. Immunolabelling with chain-specific antibodies raised against laminin α4 (*A*, *B*, *C*, *D*), α5 (*E*, *F*, *G*, *H*) and γ1 (*I*, *J*, *K*, *L*), also indicated no clear reduction in FKRP^KD^ relative to wild type at either E15.5 or P0. However, laminin β1 (*M*, *N*, *O*, *P*) showed a subtle reduction in FKRP^KD^ compared to wild type at E15.5 and P0

We next examined the impact of reduced α-dystroglycan on laminin expression. Laminin α-chains interact with dystroglycan via their LG domains although each isoform may bind with different affinities. Using chain-specific antibodies, we labelled transverse sections of the tibialis anterior and extensor digitorum longus for laminin α1, α4, α5, β1 and γ1. This showed that laminin α2 was reduced in the FKRP^KD^ relative to wild type at both E15.5 and P0 (Fig. 2b). Laminin α1 immunolabelling was only observed at the dermal–epidermal junction in the skin and myotendinous junction. Whilst no clear differences were seen in the muscle from either FKRP^KD^ or wild type with respect to laminin α1 (data not shown), expression was reduced in the skin of the FKRP^KD^ at E15.5, a feature that was less pronounced by the time of birth (Fig. 2b). These mice however, showed no other noticeable skin defects. A subtle reduction was seen with β1 in the FKRP^KD^ although this would need to be confirmed by Western blot. Immunolabelling for laminin α5, α4 and γ1 was however, equivalent to the wild type (Fig. 4). These observations imply that at these stages of development there is no compensatory increase in either laminin α4 or α5 chains which is sometimes evident in adult muscle. However, given that γ1 levels were similar in FKRP^KD^ and wild type, it remains a possibility that another α-chain at least partially compensates for the reduction in laminin α2.

### Primary myotube number and maturation is not altered in FKRP^KD^ muscle

**Fig. 5:**
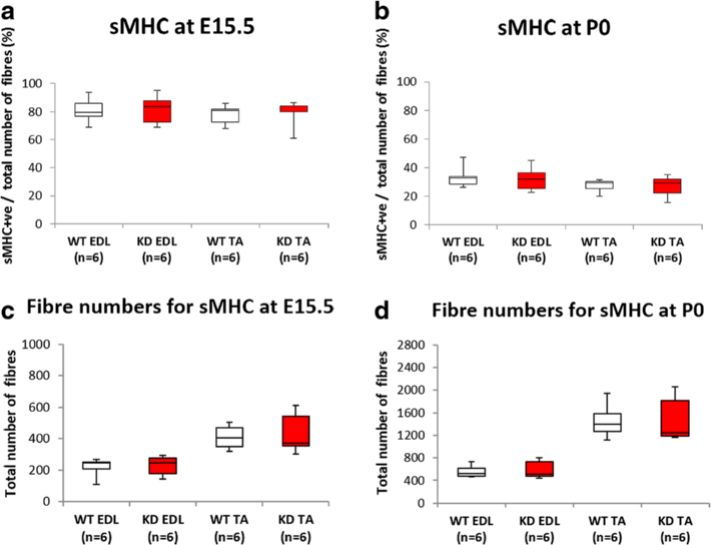
Slow myosin heavy chain expression. The majority of primary myotubes initially express slow myosin. Double labelling for slow myosin and laminin/perlecan and counts of the entire muscle enabled a comparison of the percentage of primary myotubes that expressed slow myosin in the TA and EDL at E15.5 and P0 (**a**, **b**, respectively). These analyses showed no significant differences in the percentage of slow myosin positive fibres between wild type and FKRP^KD^ indicating that primary myotube maturation was not altered by the reduction in α-dystroglycan glycosylation. The total fibre number was also not significantly altered between wild type and FKRP^KD^ (**c**, **d**). These counts were made on sections taken from the mid-third section of the hindlimb

**Fig. 6:**
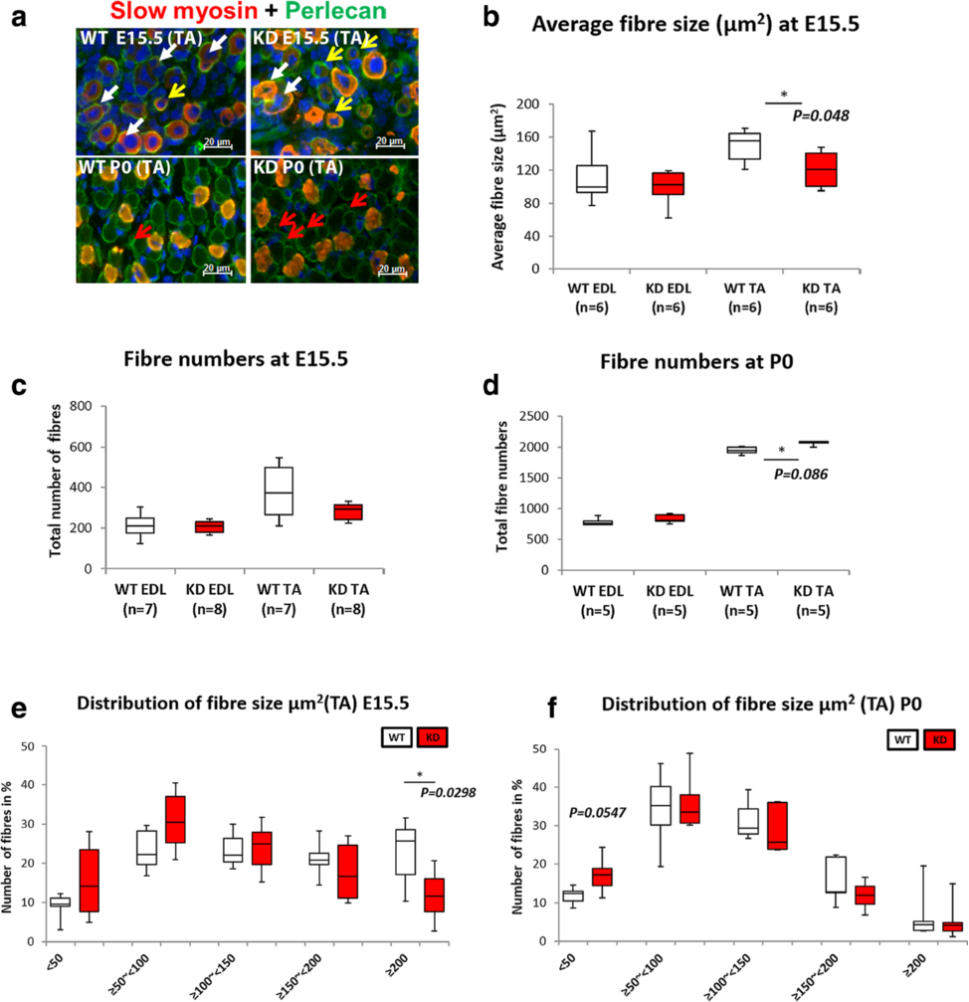
Fibre size and fibre numbers at E15.5 and P0. Double immunolabelling of slow myosin with laminin (**a**). At E15.5, many myotube clusters are outlined by perlecan immunolabelling. These clusters consist of one primary and one or more smaller secondary myotubes (*white arrow*) together with a number of secondary myotubes/fibres that have attained their own basement membrane (*yellow arrows*). Fibre size (area) therefore refers to measurements of either myotube clusters or individual myotubes/fibres that have attained their own basement membrane. There are many single myotubes enclosed within their own basement membrane in the FKRP^KD^ at E15.5 which are less prominent in the wild type (*yellow arrows*). At P0, a population of smaller myotubes/fibres were still enclosed within the primary myotube basement membrane, this was more evident in the FKRP^KD^ at P0 (*red arrows*). This was reflected by a significant reduction in fibre size in the TA muscle in the FKRP^KD^ compared to wild type (**b**). Fibre number (includes both myotube clusters and myotubes/fibres with their own basement membrane) at E15.5 was not significantly different between FKRP^KD^ and wild type as was indicated in Fig. 5 where different sections along the mid-third of the muscle length were analysed (**c**). A comparison of the size distribution graph clearly shows a shift towards smaller clusters in FKRP^KD^ relative to wild type at E15.5 attributed to a population of smaller secondaries attaining their own basement membrane (**e**). At P0, in sections taken at the midpoint region of the limb fibre counts showed a significant increase in fibre number in the TA but not in the EDL of the FKRP^KD^ compared to wild type at P0 (**d**). The size distribution graph showed this to be due to an increase (*p* = 0.0547) in number of smaller fibres in the FKRP^KD^ at P0 compared to wild type in the TA (**f**)

Primary myotubes differ from secondary myotubes in their size, number, development and expression of myosin chain isoforms at E15.5 in the mouse [38, 39]. The majority of slow myotubes initially express slow myosin [40] consequently we initially evaluated the number of slow myosin expressing myotubes in both the EDL and TA. Using this criteria there were no significant differences between FKRP^KD^ and wild type with respect to primary myotube number (Fig. 5) indicating that reduced α-dystroglycan glycosylation did not influence primary myotube numbers. However, it was formally possible that an alteration in the proportion of primary myotubes expressing slow myosin could have influenced this result, consequently we next checked this parameter. As can be seen from Fig. 5 this proportion decreased between E15.5 and P0 in both the FKRP^KD^ and wildtype as myosin composition changes with maturity depending on the muscle. Nonetheless, the proportion of slow myosin expressing myotubes was not significantly different between the FKRP^KD^ and wild type at either age. This indicates that myotube maturation as determined by slow myosin heavy chain expression was not altered by a reduction in α-dystroglycan glycosylation and reduction in laminin α2. Interestingly total fibre number in the TA was not significantly different which contrasts with measurements taken from midpoint along the limb at P0 shown in Fig. 6 where there was a significant increase in the number of smaller secondary myotubes in the FKRP^KD^ relative to wild type. Secondary myotubes initially form close to early neuromuscular junctions, this result may therefore reflect the distance these sections were from the innervation zone.

### Myotube cluster size at E15.5 is reduced but total fibre number at P0 is increased across the mid belly region of the muscle of FKRP^KD^ relative to wild type

Substantial myoblast proliferation fuels the formation of secondary myotubes. In view of the well recognized mitogenic effects of laminin we next evaluated myotube cluster size (one primary myotube and between one and two secondary myotubes enclosed within a single basement membrane) (Fig. 6a). Secondary myotubes initially form in the midbelly region of the muscle and with maturation extend the length of the muscle. In order to make a precise comparison between wild type and FKRP^KD^ mice, our measurements were therefore undertaken across the midbelly region of each muscle. At E15.5 average myotube cluster size was significantly reduced in the TA but not the EDL of the FKRP^KD^ relative to wild type (Fig. 6b). Further analysis of the size distribution of these clusters showed that in both muscles there was a shift towards smaller clusters in the FKRP^KD^ relative to wild type (Fig. 6e). Since primary myotube number was not significantly different these observations were indicative of an alteration in secondary myogenesis (Fig. 6c).

However, at P0, when many but not all secondary myotubes had attained their own basal lamina, total fibre number was significantly increased in the TA but not the EDL of the FKRP^KD^ compared to wild type at P0 (Fig. 6d). Analyses of the fibre size distribution showed this to be due to a significantly higher number of smaller fibres in the FKRP^KD^ relative to wild type in the TA. These counts were taken from sections calculated to come from a region midway between the patella and ankle joint of the hindlimb (Fig. 6f).

### FKRP^KD^ mice show a reduced number of Pax7^+^ progenitor cells in the TA muscle but not the EDL

**Fig. 7:**
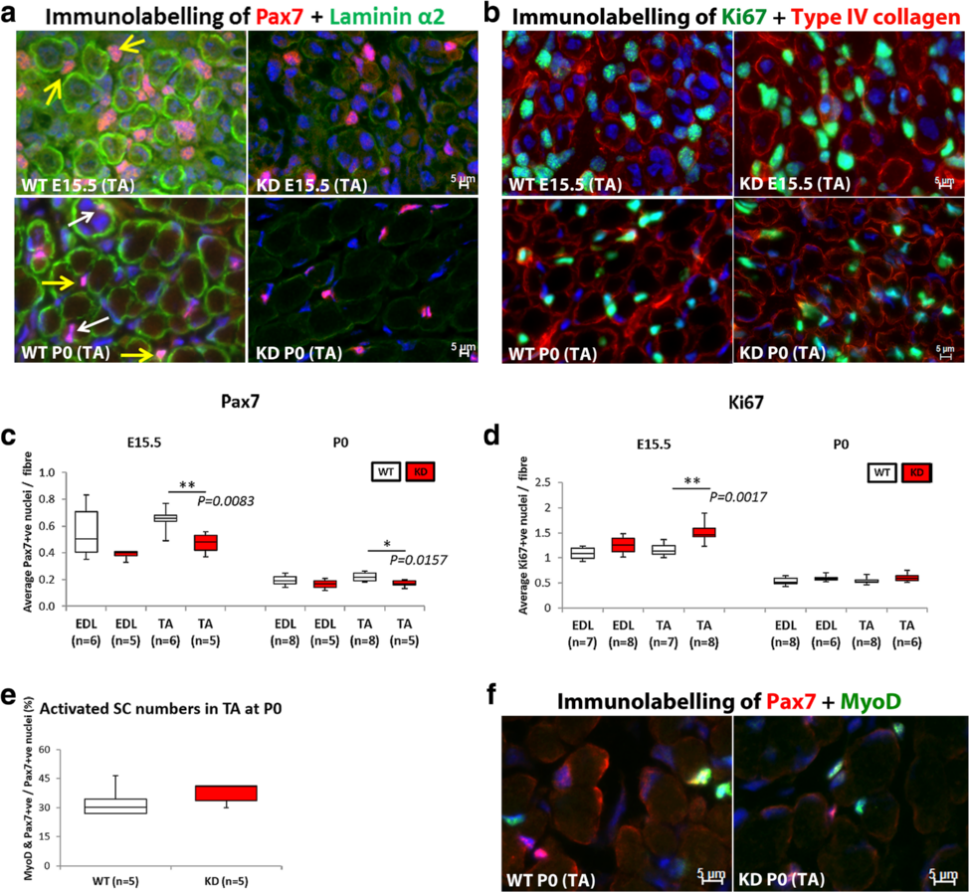
Pax7 positive progenitor cell quantification. **a** shows double immunolabelling for Pax7 and laminin α2 in the TA, whilst **b** shows Ki67+ and type IV collagen in the TA. *White arrow* indicates Pax7^+^ nuclei laying beneath the basal lamina whilst the *yellow arrow* shows Pax7^+^ nuclei outside of the basal lamina (P0). However, many nuclei were in an ambiguous position and so only, the total number of Pax7^+^ nuclei was counted. These counts expressed as the number of Pax7^+^ nuclei per fibre showed a significant reduction in the TA at E15.5 and P0 (*p* = 0.0083 and *p* = 0.0157, respectively) in the FKRP^KD^ relative to wild type (**c**). There was however, a significant increase (*p* = 0.0017) in the number of Ki67 (cell proliferation marker) positive nuclei was observed in the TA of the FKRP^KD^ at E15.5 but not at P0 (**d**). The proportion of Pax7 positive nuclei that were also expressing MyoD was not significantly different between WT and FKRP^KD^ (**e**). Immunolabelling for Pax7/MyoD is shown in (**f**). All immunolabelling was carried out on serial transverse sections

In view of the increase in myotube cluster size we next evaluated the expression of the transcription factor Pax7 which directs the differentiation of myogenic progenitor cells down the muscle lineage (Fig. 7a, b). Counts were made across the entire cross sectional area of the muscle. Quantification of the number of Pax7^+^ nuclei (expressed as the number per fibre) in the TA and EDL at E15.5 and P0 is shown in Fig. 7c where it can be seen that the number of Pax7^+^ nuclei per fibre was significantly reduced in the FKRP^KD^ TA at E15.5 and P0 (*p* = 0.0083 and *p* = 0.0157 respectively). Interestingly as with most of the other quantitative analyses there was a trend for the number of Pax7^+^ nuclei per fibre to be reduced in the EDL but this did not achieve statistical significance. Whilst the majority of Pax7^+^ nuclei appeared to be within the confines of the basal lamina at P0 - as delineated by laminin immunolabelling, there were also many whose location was ambiguous and so we could not accurately quantify whether satellite cell homing itself had been disturbed (Fig. 7a).

Interestingly the proportion of Pax7^+^/MyoD^+^ nuclei in the FKRP^KD^ TA was similar to wild type at P0 indicating that myogenic differentiation was not delayed (Fig. 7e, f). However, the number of Pax7^+^ nuclei/fibre was significantly reduced in the FKRP^KD^ TA compared to wild type.

### The number of Ki67 positive nuclei in the FKRP^KD^ was increased relative to wild type in the TA but not the EDL at E15.5

In view of the reduction in Pax7 positive nuclei per fibre we next sought to determine if cellular proliferation was altered. Labelling for Ki67 (cell proliferation marker) positive nuclei showed a significant increase (*p* = 0.0017) in the TA of the FKRP^KD^ but not the EDL at E15.5. At birth these differences were no longer evident (Fig. 7d).

### Myoblasts isolated from E15.5 embryos show reduced myogenicity relative to wild type

**Fig. 8:**
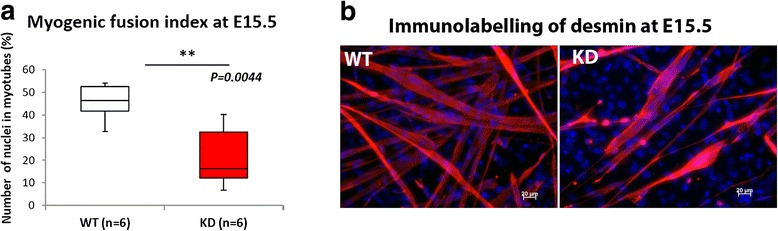
Myoblast fusion at E15.5. Myoblasts were isolated from the limbs at E15.5 for the further evaluation of reduction in cluster size that we observed on transverse sections. The myogenic fusion index calculated by counting the proportion of nuclei contained within myotubes showed a significant reduction in FKRP^KD^ relative to wild type (**a**). Desmin immunolabelling further showed that the number of myotubes that formed 4–5 days after plating on Matrigel pre-coated coverslips was reduced in the FKRP^KD^ (*n* = 6 wild type and 6 FKRP^KD^ pups) (**b**)

Since our data indicated an alteration in secondary myogenesis reflected by the reduction in cluster size, we undertook primary cell culture by isolating myoblasts at E15.5, culturing these cells on a matrigel coated substrate and allowing them to fuse. In agreement with the reduction in myotube cluster size FKRP^KD^ cells showed a significant reduction in the fusion index (Fig. 8a).

## Discussion

The secondary dystroglycanopathies are associated with a wide clinical spectrum and a proportion of patients show defects in the central nervous system in addition to a muscular dystrophy. However, whilst neuronal migration defects together with pial basement membrane defects occur prenatally, no previous reports have documented a prenatal onset for the disease in muscle. In this study we hypothesized that, in view of the pivotal role played by α-dystroglycan in basement membrane organisation via its binding to ligands such as laminin and perlecan [2], muscle development would be altered such that fibre number, size and satellite cell number would be reduced. Since we and others have previously shown that the extent of α-dystroglycan glycosylation increases with gestational age [25, 26] in both chick and human muscle we further reasoned that the alterations would be confined to the later stages of myogenesis.

Dystroglycan is essential for the initial binding of laminin on the cell surface, whereas β1 integrins and perlecan are subsequently required for laminin matrix assembly [41]. In the present study we show that organisation of the basement membrane as determined by IIH6 and laminin α2 immunolabelling is altered by E15.5 in the FKRP^KD^ mice. This corresponds to a period when myoblasts align and fuse along the surface of the primary myotube and all are enclosed within a common basal lamina [30]. Since the majority of primary myotubes label for slow myosin heavy chain during the early stages [42] we used this as a marker for primary myotube formation. Primary myotube number was not significantly different in either the TA or EDL and muscle length as determined by the length of femur and tibia was also not significantly different between the FKRP^KD^ or wild type littermates. Primary myotubes extend the full length of the muscle from their earliest stages of formation, and these observations therefore imply that early myotube formation and differentiation are not altered by defects in α-dystroglycan which are nonetheless present by this stage. Indeed the proportion of slow myosin heavy chain positive myotubes was not significantly different between wild type and FKRP^KD^ and the expression patterns of fast MHC and developmental MHC was also the same between the two groups at both E15.5 and P0 which further suggested no obvious delay in maturation (data not shown).

Myotube clusters are a characteristic of muscle development mid gestation and they usually consist of a single primary surrounded by one or more (depending on the muscle) secondaries. Cluster size was found to be significantly reduced in the TA but not the EDL at E15.5. Since this parameter also showed a reduction in the EDL which was not statistically significant we believe that this time point represents the initial point of divergence in the hindlimb muscles. The isolation of myoblasts from E15.5 hindlimbs showed a reduction in the fusion index which together with the reduction in cluster size suggests that a reduction in α-dystroglycan glycosylation impacts upon secondary myotube formation. Interestingly the number of Ki67 positive proliferating cells in the TA was increased at E15.5 but not P0 suggesting a transitory increase in the division of cells not yet committed to the myogenic lineage. Given that fibre number was slightly increased in the FKRP^KD^ at birth we assume that these cells do subsequently contribute to fibre formation.

α-dystroglycan binds to LG domain containing proteins of the extracellular matrix, including laminin and perlecan. At E15.5 and P0, immunolabelling of laminin α2 showed a reduction in FKRP^KD^ mice relative to controls. However, there was no apparent alteration in perlecan despite previous data showing the existence of a trimolecular arrangement between α-dystroglycan, perlecan and laminin, with perlecan binding to α-dystroglycan indirectly [43]. This suggests that the incorporation of laminin into the basement membrane during development is more dependent on α-dystroglycan glycosylation than perlecan, indeed the latter may have other binding partners that are sufficient to facilitate its incorporation. Labelling for type IV collagen (data not shown) displayed no clear difference in FKRP^KD^ mice relative to controls at either E15.5 or birth showing that the other aspects of the basement membrane were unaltered by the changes in α-dystroglycan glycosylation. This is in contrast to the observations made in dystroglycan-null embryoid bodies where the expression pattern of type IV collagen has been shown to be disrupted [44]. This may reflect tissue-specific differences or may reflect the absence of both α and β-dystroglycan in these cells.

Fibre number at birth showed a significant increase in the FKRP^KD^ TA but not EDL. This may be interpreted in several ways namely that there is a more rapid breakup of the clusters due to a weakened basement membrane or alternatively an increase in branching of the secondary myotubes. Branching is normally a response to repeated injury/overloading [45]; however, this has also been reported in dystrophin-deficient mdx and caveolin-3-deficient mouse muscle during development [46]. Indeed, it is possible that the lack of α-dystroglycan glycosylation and the reduction in laminin α2 deposition provide inappropriate cues for the early secondary and tertiary myotubes and they branch.

Immunolabelling using an antibody to pan laminin showed no apparent difference between the two groups at both time points (Fig. 2b). Since pan laminin recognises several laminin chains including β1 and γ1, this observation strongly suggested that there might be a partial compensation by other laminin isoforms prior to birth for the loss of laminin α2. Laminin α5 is often upregulated in neuromuscular conditions and thought to compensate for other α-chains. However, labelling with antibodies to laminin α4, α1, α5 and γ1 failed to detect any significant differences between FKRP^KD^ and controls. β1 on the other hand showed a subtle reduction suggesting that the expression of laminin β1 was rather influenced by the loss of laminin α2 and implies that β2 may partially compensate. However, real-time PCR indicated no significant differences between FKRP^KD^ and wild type, although it was noted that there was a trend for all to be reduced in the FKRP^KD^ at E15.5. Indeed, it could be that a reduction in expression levels of ligand proteins is only manifested later on during development when there has been a long-term reduction in the relevant receptor.

Satellite cells are first evident around E17 and typically identified using the paired box transcription factor, Pax7. We have therefore referred to those nuclei expressing Pax7 as progenitor cells rather than satellite cells. Whilst they are traditionally thought to be derived from the somites [47], there is now evidence to suggest that some adult satellite cells may have alternative origins other than the dermomyotome-derived Pax3+/Pax7+ progenitor cells [48]. The dynamic interaction between the extracellular matrix and the satellite cell plays a key role in determining activity [48, 49] and in agreement with this, we have previously shown the satellite cell niche to be disturbed in the dystroglycanopathies [50]. A series of transcription factors drive the progression from quiescence to activation, proliferation, and differentiation/self-renewal. Quiescent satellite cells are characterised by their expression of Pax7 and Myf5 but not MyoD or Myogenin. The initiation of differentiation and fusion is marked with the expression of Myogenin, which in concert with MyoD will activate muscle-specific genes [48]. In the present study, we observed that the proportion of activated progenitor cells (Pax7+/MyoD+) was not significantly different in the TA between FKRP^KD^ and wild type at P0. This indicates that the differentiation itself has not been delayed as is supported by the pattern of myosin heavy chain expression. However, the absolute number of Pax7 positive cells was decreased in the FKRP^KD^ TA relative to the wild type indicating that the number of committed progenitor/satellite cells has been reduced in the mutant which would be expected to compromise not only postnatal growth but also the regenerative capacity of the muscle.

Whilst there has been no previous detailed quantitative assessment of muscle development in secondary dystroglycanopathy mice, other work has suggested that the severity of the phenotype may correlate with when the expression of genes, such as that encoding for fukutin, are deleted [35, 51]. Indeed, two groups previously compared the two distinct conditional fukutin knock-out (cKO) mice; the first, a myofibre-selective fukutin-conditional knock-out (using the muscle creatine kinase promoter to drive the deletion), which showed a mild muscular dystrophy whilst the second, deleted fukutin in muscle precursor cells using the Myf5 promoter. The earlier deletion resulted in a mouse with a more severe form of muscular dystrophy. In the present paper assuming that fukutin and FKRP have a similar function, the reduction in the number of Pax7 progenitor cells identifies a possible mechanism behind previous observations of a regeneration disorder [35, 51]. Another interesting aspect is the role of the CT carbohydrate antigen {GalNAcβ1,4[NeuAcα2,3]Galβ1(-3GalNAc or -4GlcNAc)} on α-dystroglycan which when expressed outside its usual synaptic location leads to an accumulation of satellite cells, reduced myofibre size and altered neuromuscular junctions [52]. This work would seem to indicate as did the work of Ross et al. [50] that alterations in the glycosylation pattern of α-dystroglycan can directly impact upon the stem or satellite cell population with clear implications for postnatal muscle growth.

## Conclusions

In summary, these data show for the first time a prenatal onset with regard to the disease process and identify an early reduction of laminin α2, reduction of myogenicity and depletion of Pax7 positive progenitor cells which would be expected to compromise subsequent postnatal muscle growth and its ability to regenerate postnatally. These findings are of significance to the development of future therapies in this group of devastating conditions.

## Abbreviations

DAGC: dystrophin-glycoprotein complex
EDL: extensor digitorum longus
EDTA: ethylene diamine tetra acetic acid
FKRP: fukutin-related protein
FKRP^KD^: FKRP knock-down
LARGE: like-acetylglucosaminyltransferase
LGMD2I: Limb-girdle muscular dystrophy type 21
MDC1C: congenital muscular dystrophy type 1C
MEB: muscle-eye-brain disease
TA: tibialis anterior
WWS: Walker–Warburg syndrome
